# Distinct colicin M-like bacteriocin-immunity pairs in *Burkholderia*

**DOI:** 10.1038/srep17368

**Published:** 2015-11-27

**Authors:** Maarten G. K. Ghequire, René De Mot

**Affiliations:** 1 Centre of Microbial and Plant Genetics, University of Leuven, 3001 Heverlee, Belgium

## Abstract

The *Escherichia coli* bacteriocin colicin M (ColM) acts via degradation of the cell wall precursor lipid II in target cells. ColM producers avoid self-inhibition by a periplasmic immunity protein anchored in the inner membrane. In this study, we identified *colM*-like bacteriocin genes in genomes of several β-proteobacterial strains belonging to the *Burkholderia cepacia* complex (Bcc) and the *Burkholderia pseudomallei* group. Two selected *Burkholderia ambifaria* proteins, designated burkhocins M1 and M2, were produced recombinantly and showed antagonistic activity against Bcc strains. In their considerably sequence-diverged catalytic domain, a conserved aspartate residue equally proved pivotal for cytotoxicity. Immunity to M-type burkhocins is conferred upon susceptible strains by heterologous expression of a cognate gene located either upstream or downstream of the toxin gene. These genes lack homology with currently known ColM immunity genes and encode inner membrane-associated proteins of two distinct types, differing in predicted transmembrane topology and moiety exposed to the periplasm. The addition of burkhocins to the bacteriocin complement of *Burkholderia* reveals a wider phylogenetic distribution of ColM-like bacteriotoxins, beyond the γ-proteobacterial genera *Escherichia, Pectobacterium* and *Pseudomonas*, and illuminates the diversified nature of immunity-providing proteins.

*Burkholderia* is a common soil inhabitant and exhibits attractive properties such as degradation of environmental pollutants, production of bioactive secondary metabolites and biocontrol of phytopathogenic fungi^1^,^2^,^3^. Conversely, this β-proteobacterial genus includes phytopathogenic species and some members also pose a major threat to animal and human health. The *Burkholderia pseudomallei* group includes *B. pseudomallei*, the causative agent of melioidosis, and *Burkholderia mallei*, giving rise to glanders^4^,^5^. Another group, comprising several phylogenetically closely related *Burkholderia* species, constitutes the *Burkholderia cepacia* complex (Bcc) and these opportunistic pathogens frequently colonize lungs of cystic fibrosis and immunocompromised patients^2^,^6^,^7^. Therapeutic options against these pathogens are scarce, urgently requiring the development of novel antibacterials. Of particular interest are antagonistic compounds with narrow-spectrum of activity in order to minimize collateral damage to beneficial microbiota.

One possible strategy to identify new *Burkholderia*-specific therapeutics is to exploit the antibacterial arsenal encoded in *Burkholderia*’s own genomes. Evidence for specific antagonism between *Burkholderia* strains has been reported^8^,^9^, but few compounds mediating such interactions have been characterized: enacyloxin, a polyketide antibiotic from *Burkholderia ambifaria* AMMD^10^; capistruin, a ribosomally encoded lasso peptide from *Burkholderia thailandensis* E264^11^; the plant lectin-like protein LlpA from *Burkholderia cenocepacia* AU1054^12^; and a tailocin complex from *B. cenocepacia* BC0425^13^,^14^. Another mechanism mediating antibiosis is contact-dependent inhibition, demonstrated in *B. thailandensis* and *B. pseudomallei*. There, toxic domains are directed to target cells via two-partner secretion^15^,^16^.

Contrary to *Burkholderia*, the genus-specific bacteriotoxic armamentarium of γ-proteobacteria has been studied in much more detail, with colicins from *Escherichia coli*^17^ and pyocins from *Pseudomonas aeruginosa* serving as prominent model systems^18^. Colicin M (ColM) is one such well-characterized bacteriocin taking advantage of the FhuA outer membrane siderophore transporter as a receptor and highjacking the TonB/ExbB/ExbD machinery for further translocation via an amino-terminal TonB-interacting box^19^,^20^. Inhibitory action is mediated by phosphatase activity that cleaves lipid II peptidoglycan precursors, releasing undecaprenol and pyrophosphoryl groups^21^,^22^. Immunity is provided by a downstream-located gene, transcribed in opposite direction, that encodes a cytoplasmic membrane-anchored immunity protein (ColM immunity protein, Cmi), facing the periplasmic space and protecting the producer cells via an unknown mechanism^23^,^24^. The Cmi monomer is composed of four β strands and four α helices and requires an intramolecular disulfide bond to be functional^25^. Interestingly, Usón *et al.*^26^ reported dimerization via domain swapping of a significant fraction of Cmi proteins, stabilization between the two monomers being provided by two intermolecular disulfide bridges. The Cmi fold is exemplary for YebF family proteins that require OmpF for their secretion^27^. YebF appears as a monomer with an intramolecular disulfide bond.

ColM-like bacteriocins have also been characterized in two other γ-proteobacterial genera: *Pseudomonas*^28^,^29^,^30^ and *Pectobacterium*^31^. A putative immunity gene sharing homology with *cmi* was predicted for pectocin M1 in *Pectobacterium carotovorum* PC1, showing the same gene organization as *colM*-*cmi*^31^. To date, no *cmi*-like immunity genes have been retrieved in *Pseudomonas*^30^.

In this work we purified and characterized two ColM-like proteins from the Bcc species *B. ambifaria*, killing target cells with genus-specific activity. These proteins display a similar domain composition as γ-proteobacterial ColM-type bacteriocins although their catalytic domains are poorly conserved. The toxin genes are associated with one of two novel types of genes which provide bacteriocin-specific immunity.

## Results

### ColM-like bacteriocin genes in *Burkholderia* genomes

**Figure 1:**
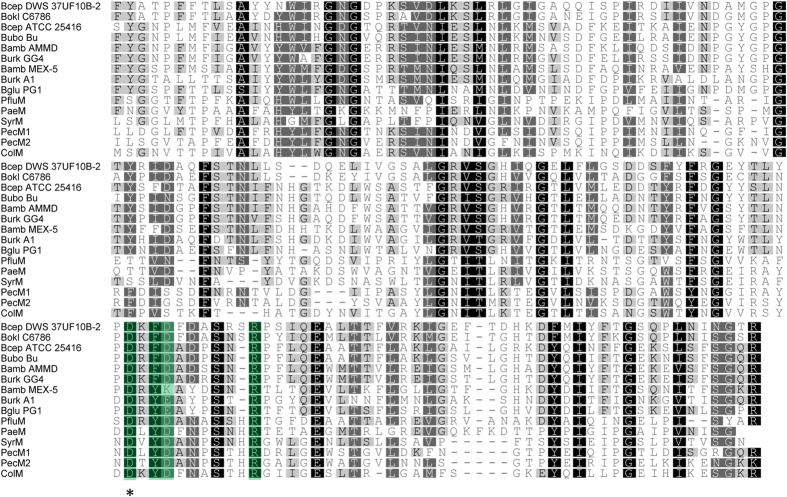
Multiple sequence alignment of ColM domains in selected burkhocins and characterized γ-proteobacterial bacteriocins. Abbreviations for species names: Bamb, *Burkholderia ambifaria*; Bcep, *Burkholderia cepacia*; Bglu, *Burkholderia glumae*; Bokl, *Burkholderia oklahomensis*; Bubo, *Burkholderia ubonensis*; Burk, *Burkholderia* sp. Previously characterized ColM domain-carrying bacteriocins are *E. coli* ColM, *Pectobacterium* PecMs, *P. aeruginosa* PaeM, *P. fluorescens* PfluM, and *P. syringae* SyrM. Shading reflects the degree of conservation. The conserved residues in the catalytic site are shaded in green. The aspartate residue that was mutated in BurM2 is marked with an asterisk.

**Figure 2:**
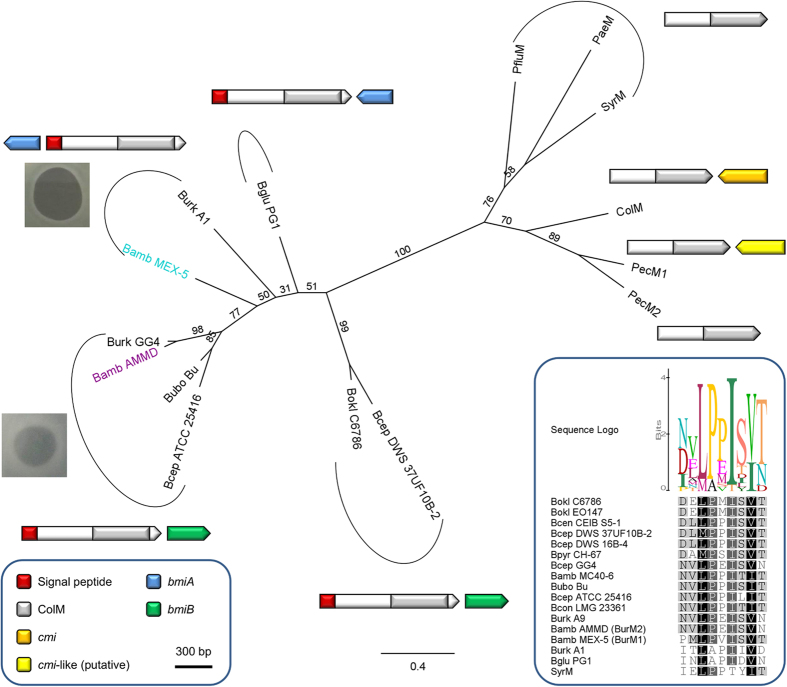
Phylogeny of ColM domains in burkhocins and γ-proteobacterial bacteriocins. Maximum likelihood phylogenetic tree of ColM domains of selected *Burkholderia* BurMs, *E. coli* ColM, *Pectobacterium* PecMs, *P. aeruginosa* PaeM, *P. fluorescens* PfluM, and *P. syringae* SyrM. *Burkholderia* species abbreviations are the same as in Fig. 1. Bamb MEX-5 (BurM1) and Bamb AMMD (BurM2) are shown in teal and purple, respectively. The bacteriocin activity of BurM1 against LMG 18829 (clear halo) and of BurM2 against LMG 18943 (turbid halo) in spot assays is illustrated. Scale bar represents 0.4 substitutions per site. Bootstrap values (percentages of 1000 replicates) are shown at the branches. Schematic gene organizations of bacteriocins and (putative) associated immunity genes are displayed next to the clades or branches (delimited by arcs). The arrows correspond with the gene orientations. For pectocin M2 and pseudomonad ColM-like bacteriocins, no adjacent immunity genes have been described. The color legend (box with scale bar, bottom left) describes the indicated domains and signal peptide sequences. Predicted signal sequences in the immunity genes, if present, are not shown. Sequence alignment of the conserved region following the signal sequence in BurMs and in SyrM is shown as inset (bottom right). Shading reflects the degree of conservation. The sequence logo graph visualizes the degree of consensus for each position.

Genomic homology searches in the β-proteobacterial genus *Burkholderia,* using ColM domains (Pfam PF14859) of functionally characterized ColM-type bacteriocins from *E. coli, Pectobacterium* and *Pseudomonas* as queries, revealed several uncharacterized proteins encoded by 16 strains (Table 1). These proteins almost exclusively occur in Bcc strains, although two representatives were detected in *Burkholderia oklahomensis*, member of the Pseudomallei group. Among these proteins, further termed burkhocin M (BurM), the catalytic domain displays considerable sequence divergence, with ~55% pairwise identity, and only borderline homology with the γ-proteobacterial domains (~23% pairwise identity) is apparent (Fig. 1). The *Burkholderia* ColM modules are typified by a carboxy-terminal extension of 26–36 AA, that is absent from γ-proteobacterial representatives and not part of the Pfam ColM domain (Fig. S1). Based on phylogenetic analysis of the ColM domains (Fig. 2), the BurMs constitute different subgroups, coinciding with the extent of homology between both their respective amino-terminal and carboxy-terminal parts (Table 1, Fig. S1). Similarly to ColM and M-type pyocins, the BurMs equally lack a defined domain in their amino-terminal part. These *Burkholderia* proteins are further typified by a cleavable amino-terminal secretion signal sequence: for 12 strains, a Tat signal peptide is predicted with high probability (PRED-TAT reliability scores >0.96), whereas a potential Sec signal peptide is likely present in *B. ambifaria* AMMD (score 0.917) and *Burkholderia* sp. A1 (score 0.956). Only two proteins (nearly identical sequences of *B. cepacia* GG4 and *Burkholderia* sp. A9) are not classified as substrates for the Tat or Sec translocation systems (scores <0.6). A Sec-type signal sequence was also observed for *Burkholderia* and *Xanthomonas* LlpA bacteriocins^12^,^32^, whereas this is absent from most pseudomonad counterparts^33^,^34^. At the amino-terminal end of the predicted mature BurM proteins, a short conserved sequence appears (Fig. 2, alignment with sequence logo). Although not sharing homology with colicin M’s TonB box, the high degree of conservation suggests that this region may equally represent a binding box for interaction with the *Burkholderia* TonB orthologue. The presence of a similarly positioned semi-conserved motif in syringacin M (Fig. 2) is required for its anti-pseudomonad activity^28^.

The genomic context of *burM* genes is not conserved but synteny is apparent within some subsets. In the clade comprising BurMs from strains AMMD, GG4, ATCC 25416, and Bu (Fig. 2), toxin genes are intertwined between genes encoding glutamate synthase and an amino acid symporter. In contrast, in the *B. cepacia* strains DWS 37UF10B-2 and DWS 16B-4, and *B. cenocepacia* CEIB S5–1, *burM* genes are located between a LysR family regulator and a MFS transporter. The alien nature of *burM* genes, that further seem to be unique in β-proteobacterial genera, is also underlined by an aberrant GC content. Whereas the *Burkholderia* strains have a genomic GC content of 70–75%, *burM* genes reside in ~2.2 kb stretches with a GC content of only 45–55%. In *Pseudomonas*, it was found that ColM-like bacteriocins may be present as cargo genes in tailocin clusters^35^,^36^, but this does not seem to be the case for the burkholderial isolates.

### Purification and activity of burkhocins

Based on their disparate position in the phylogenetic tree and the different nature of associated putative immunity genes (see further), candidate M-type burkhocin genes *burM1* (*B. ambifaria* MEX-5) and *burM2* (*B. ambifaria* AMMD) were cloned in expression vector pET28a(+), generating pCMPG6228 and pCMPG6229, respectively. Fusions were created without the predicted secretory signal sequences to encode C-terminal His-tags, separated from the mature bacteriocins by a LE (Leu-Glu) linker, in order to avoid potential interference with the (presumed) translocation function of the conserved amino-terminal stretch. Following expression of *burM1* and *burM2* in *E. coli* BL21(DE3), recombinant proteins were purified by affinity chromatography on Ni-NTA and polished by gel filtration (Fig. S2). Identity of the purified proteins was verified via Edman degradation, yielding the predicted amino-terminal sequences of mature BurM1 and BurM2 proteins.

Next, the recombinant BurMs were tested for their antagonistic potential against a panel of 44 Bcc strains by spotting purified bacteriocin onto a lawn of the indicator bacteria of interest. Antibacterial activity for BurM1 was observed for ~26% of the strains tested, whereas only ~7% of them were killed by BurM2 (Table S1), all of the latter set being susceptible to BurM1 as well. The lower percentage of BurM2 indicators identified may reflect strain bias in the test panel. In the case of BurM2, clear halos were obtained for only one strain (*B. cenocepacia* LMG 18829), whereas almost all BurM1-generated halos were clear (Fig. 2). Turbid halos may originate from unfavorable expression conditions for the bacteriocin receptor at the cellular surface or poor receptor affinity. BurM1 and BurM2 were also tested against a selection of *Pseudomonas* isolates, but no strain proved to be susceptible (data not shown).

Via site-directed mutagenesis, a number of key residues (D226, Y228, D229, H235, and R236) were identified in the phosphatase domain of ColM^22^,^37^,^38^,^39^. These surface-exposed residues also occur in γ-proteobacterial ColM-like bacteriocins and likely facilitate binding of the targeted peptidoglycan precursors^28^,^29^,^31^. The equivalents of ColM residues D226 and R236 are strictly conserved in the BurM proteins (Fig. 1). Semi-conserved residues appear for Y228 (mainly F) and D229 (mainly D), whereas a corresponding histidine is absent. To assess the significance of this conserved stretch (sequence DKFDADSSNR in BurM2) for burkhocin activity, we constructed a BurM2 mutant in which Asp297 (residue underlined) is replaced by Ala. After expression in *E. coli* and subsequent purification of the His-tagged protein (Fig. S2), this mutant form was tested for antagonistic activity against BurM2-susceptible strains. As expected, for all indicator strains tested, BurM2 activity was lost (data not shown).

### Two sets of putative immunity genes coupled to burkhocin M genes

**Figure 3:**
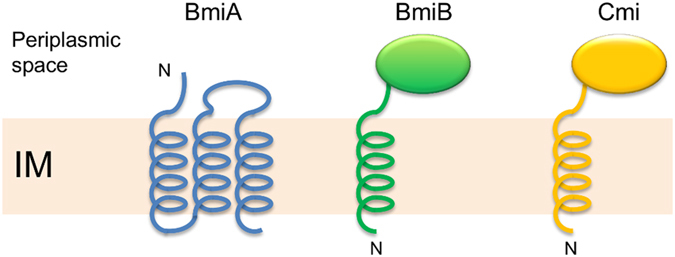
Membrane topology of Bmi proteins from *Burkholderia* spp. and Cmi from *E. coli*. BmiA carries three potential transmembrane regions, whereas BmiB, like Cmi, only has one that would span the inner membrane (IM). In the case of certain BmiB proteins, the depicted transmembrane region may actually represent a Sec-dependent cleavable signal sequence. “N” denotes the amino-terminal end of the polypeptides.

In addition to the *burM* genes, the low GC-content regions consistently encompass an infrequently annotated second open reading frame that may represent an immunity determinant. These associated genes (*bmi*, burkhocin M immunity) are either located upstream or downstream of *burM*, in opposite (upstream/downstream) or same (downstream) orientation (Table 1, Fig. 2). The encoded gene products are of two different types (Fig. 3). One set of shorter proteins (BmiA group, ~106 AA), including the *B. ambifaria* MEX-5 polypeptide, harbors three predicted transmembrane helices and lacks a cleavable signal sequence for export (Sec or Tat) (Fig. S3). A cysteine residue in the second helix is strictly conserved but additional cysteines present (for instance three in the MEX-5 BmiA protein) are not. The topology prediction implies that only a short loop connecting the second and third helix would be exposed to the periplasmic space. A second type (BmiB group, ~126 AA), being more abundant, carries a single hydrophobic helix near the amino-terminus (Fig. S4). In about half of these (such as the *B. ambifaria* AMMD protein), this segment may serve as a Sec-signal peptide and be cleaved off (PRED-TAT reliability scores >0.94). Both types lack the YebF domain signature (Pfam PF13995) characteristic for Cmi and structurally related YebF-like proteins. However, despite the absence of primary sequence homology, members of the BmiB group would adopt the general Cmi/YebF topology of periplasmic proteins with an amino-terminal cytoplasmic membrane anchor. Moreover, the presence of two perfectly conserved cysteine residues in the *Burkholderia* proteins advocates functional similarity with the Cmi immunity protein. Since the replacement of the Cmi membrane anchor by a cleavable signal peptide yields a soluble periplasmic variant that retains protective activity^23^, it is possible that the BmiB proteins function in the periplasm either as membrane-anchored or as soluble proteins. The phylogenetic tree of this second group displays a similar clade topology as observed for the ColM domains of the BurMs (Fig. 2, Fig. S4). This is reminiscent of the coevolution observed between nuclease domains and cognate immunity proteins of S-type pyocins^18^ and it supports the hypothesis that these genes may encode cognate immunity proteins.

Notably, both sets of putative immunity genes also occur as ‘orphan’ genes, at a similar – without associated *burM* – or at different genomic location in some *Burkholderia*. Orphan *bmiA* genes are very common among strains of the Pseudomallei group, although associated *burM* genes could not be identified in our study. On the other hand, Bcc strains frequently encode orphan *bmiB* genes. Such orphans can provide a reservoir of immunity and may trap trespassing bacteriocins.

### Burkhocin M activity is impeded by expression of a cognate immunity gene

Associate genes of *burM1* (*bmiA*_*MEX5*_) and *burM2* (*bmiB*_*AMMD*_, with predicted Sec-signal peptide) cloned in shuttle vector pJB3Tc20 (pCMPG6231 and pCMPG6232, respectively) were electroporated to BurM-susceptible strains LMG 18830, LMG 18941, LMG 18943, and LMG 24506, and tested for BurM sensitivity via spot assay, using strains with empty vector as controls (Table 2). Indicator strain LMG 18829 was not suitable since it is resistant to high concentrations of tetracycline used as selective marker. Strains equipped with *bmiA* became fully immune to BurM1 activity at 1 mg/ml, whereas those with *bmiB* acquired immunity to BurM2, at equimolar test conditions. On the contrary, *bmiA* was not able to provide immunity to BurM2, and *vice versa*, suggesting that both sets of BurM-associated genes act as cognate immunity partners.

## Discussion

*Burkholderia* strains host the biosynthetic machineries for a plethora of biologically active secondary metabolites, including diverse molecules mediating antibiosis. Conversely, relatively few ribosomally encoded antimicrobial peptides and proteins have been identified in this genus^9^,^11^,^12^,^13^,^15^,^16^. In this study we characterized two members of a novel bacteriocin family (burkhocin M) in *Burkholderia.* Based on features shared with the toxins and immunity proteins of the ColM family occurring in γ-proteobacteria, it is anticipated that these bacteriocins affect integrity of the β-proteobacterial cell envelope by interfering with peptidoglycan biosynthesis. This is supported by the observation that the mutation of a conserved Asp residue is pivotal for obtaining a functional burkhocin M, in line with previous results for ColM and ColM-like bacteriocins in γ-proteobacteria. Nevertheless, the “core” residues likely interacting with lipid II or a related peptidoglycan precursor are not fully conserved, which may reflect differences in substrate specificity. In this respect, it will be of interest to elucidate whether the carboxy-terminal extension, that appears to be specific for the β-proteobacterial members of the ColM family, plays a role in this interaction or would have a different function, for instance in receptor recognition.

Unexpectedly, we identified two independent strategies of burkhocin M immunity in *Burkholderia*. The predominant BmiB-type proteins display a similar membrane topology as Cmi, with one transmembrane helix and a periplasmic domain, and also carry a conserved cysteine pair. Despite the lack of primary sequence homology with Cmi, this suggests that BmiB proteins may adopt a similar structure as Cmi and inhibit the cognate burkhocins in a comparable manner as in *E. coli*, although the exact Cma-Cmi immunity mechanism yet remains to be revealed. The less abundant BmiA-type proteins represent a novel type of immunity-providing protein, predicted to contain three transmembrane helices and a short loop exposed to the periplasmic space. The BmiA proteins carry multiple cysteines but only one position appears to be perfectly conserved. Potentially, such residue may enable homodimer formation by analogy with the cysteine-mediated dimerization observed for Cmi^26^. Another interesting issue to scrutinize is how protection can be provided by the integral BmiA-type proteins. Here, focus would be on the role of the periplasm-exposed loop that is envisaged to interact with a burkhocin.

Following the identification of a lectin-like bacteriotoxin^12^, this new bacteriocin family further adds to the diversity of these narrow-spectrum antibacterials, and constitutes another example of *Burkholderia* bacteriocins that are shared with γ-proteobacteria.

## Materials and Methods

### Bacterial strains and media

*Burkholderia* strains (Belgian Co-ordinated Collections of Micro-organisms) used in this study are listed in Table S1. *Burkholderia* and *E. coli* strains were routinely grown in LB broth (2.5%, MP Biomedicals) at 37 °C, with shaking at 200 rpm. Media were solidified with agar (1.5%, Invitrogen) and supplemented with filter-sterilized isopropyl-β-D-thiogalactopyranoside (IPTG, 25 μg/mL, ForMedium), ampicillin (100 μg/mL, Sigma-Aldrich), kanamycin (50 μg/mL, Sigma-Aldrich), or tetracycline (150 μg/mL, Sigma-Aldrich) when required. Bacterial strains were kept on plate at 4 °C, or stored at −80 °C in glycerol (25% v/v, Sigma-Aldrich).

### DNA methods and plasmid construction

Genomic DNA from *Burkholderia* spp. was isolated using the Puregene Yeast/Bact. Kit B (Qiagen). Genes encoding putative bacteriocins in *Burkholderia ambifaria* were amplified by polymerase chain reaction with Q5 DNA polymerase (BIOKÉ), with a C1000 Thermal Cycler (Bio-Rad), using genomic DNA as a template. PCR amplicons were purified with the QIAquick PCR Purification Kit (Qiagen), digested with NcoI/XhoI (Roche Diagnostics), ligated in pET28a(+) using T4 DNA ligase (Invitrogen), and transformed to *E. coli* TOP10F’. Transformants were verified for the presence of inserts by PCR with *Taq* polymerase (BIOKÉ). Primers used for PCR amplification are listed in Table S2. Insert-confirmed plasmids were collected with the QIAprep Spin Miniprep Kit (Qiagen) and validated by sequencing (GATC Biotech, Constance, Germany). Bacteriocin genes were cloned without their predicted amino-terminal secretory signal sequences, with the His_6_-tag located at the carboxy-terminus (terminal sequence LEHHHHHH). Constructed plasmids were pCMPG6228 (BamMEX5DRAFT_6664 from *B. ambifaria* MEX-5, GenBank EDT37562; BurM1) and pCMPG6229 (BAMB_RS01670 from *B. ambifaria* AMMD, GenBank ABI85884; BurM2). In the case of BAMB_RS01670, the gene was first cloned in pUC18, using primers PGPRB-10167 and PGPRB-10168 (digested with PstI and XbaI, Roche Diagnostics). The internal NcoI restriction site was removed via DpnI-mediated (Roche Diagnostics) site-directed mutagenesis, using PGPRB-10117 and PGPRB-10118. Sequence-confirmed plasmid DNA was used as a template for amplification and subsequent pET28a cloning, as described above. A D297A mutant (position according to numbering in full-length precursor) in BurM2 was equally constructed via DpnI-mediated site-directed mutagenesis (primers in Table S2), using pCMPG6229 as a template, resulting in pCMPG6230. Sequence confirmed pET expression plasmids were transformed to *E. coli* BL21(DE3) via heat shock transformation.

Similarly, putative immunity genes from *B. ambifaria* MEX-5 (BamMEX5DRAFT_6663, GenBank EDT37561) and *B. ambifaria* AMMD (BAMB_RS01665, GenBank AJY23231) were PCR amplified (including the respective upstream ribosome binding sites), digested with PstI/XbaI and SphI/BamHI, respectively, ligated in shuttle vector pJB3Tc20, transformed to *E. coli* TOP10F’ and insert verified via sequencing. Resulting plasmids were pCMPG6231 and pCMPG6232, respectively. All plasmids used in this study are listed in Table S2.

### Overexpression and purification of recombinant *B. ambifaria* BurM proteins

Recombinant His-tagged BurM1 and BurM2 were generated in *E. coli* BL21(DE3) carrying the plasmids pCMPG6228 and pCMPG6229, respectively. 1-mL volumes of overnight cultures were transferred to 500 mL LB and incubated at 37 °C until an OD_600_ of 0.7. Then IPTG was added at a final concentration of 1 mM and cultures were incubated at 20 °C for 16 hours, shaking at 200 rpm. Next, cells were harvested in a Beckman X-15R (20 min, 5000 *g*) and kept overnight at −20 °C. Following day, cell pellets were thawed, resuspended in lysis buffer (300 mM NaCl, 50 mM NaH_2_PO_4_, 10 mM imidazole, pH 8.0) and disrupted by sonication using a Branson Digital Sonifier 250 (amplitude 18%, 8 cycles of 30 sec on/off). Next, protein extracts were treated with benzonase (0.01 U/μL, 37 °C, 1 hour; Sigma-Aldrich) and cleared via centrifugation (25 min, 10000 *g*) and filtering (0.25 μm, MilliPore). Presence of recombinant proteins was verified by SDS-PAGE and subsequent coomassie blue staining. Next, protein extracts were applied on a 5-mL HisTrap column (GE Healthcare) and purified by nickel affinity chromatography, using an Äkta Purifier (GE Healthcare). Bound proteins were eluted with a linear gradient of 10–500 mM imidazole in lysis buffer and verified for purity by SDS-PAGE. Selected elution fractions were dialyzed to TRIS buffer (20 mM, 200 mM NaCl, pH 7.5) and polished by gel filtration chromatography on a Superdex 200 pg 16/60 column (1 ml/min, GE Healthcare), using the TRIS buffer as running buffer. The same expression and purification procedure was followed for the generation of BurM2 mutant protein.

The concentration of purified proteins was estimated by absorbance measurement at 280 nm with a Spectronic Genesys 6 (Thermo). Molar extinction coefficients were 45,840 mol/L^−1^ cm^−1^ for BurM1 (37,663 Da), 49,975 mol/L^−1^ cm^−1^ for BurM2 (37,626 Da) and 49,975 mol/L^−1^ cm^−1^ for the D297A mutant protein (37,582 Da)^40^. Amino-terminal amino acid (AA) sequences of PVDF-blotted recombinant proteins were determined via Edman degradation, using a Procise 491 cLC protein sequencer (Applied Biosystems).

### Bacteriocin spot assay

Antagonistic activity of purified recombinant proteins was determined by a spot assay test. LB plates were overlaid with 5 mL of soft agar (0.5%), seeded with 50 μL of an indicator culture (16 hours, ~10^9^ CFU/ml) to generate a bacterial cell lawn. 10-μL spots of purified recombinant protein (20 μM) were applied on the plates. TRIS buffer was used as a negative control. After drying, plates were incubated overnight at 37 °C and scored the following day for the presence of zones of growth inhibition (halos).

### Electroporation of pJB3Tc20-derived plasmids to *Burkholderia* spp

Electrocompetent *Burkholderia* cells were prepared as described previously with minor modifications^41^. Briefly, 1-mL volumes of overnight cell cultures were centrifuged (3 min, 5000 *g*), supernatants removed and cells washed twice with 1 mL of 300 mM sucrose (VWR International), and finally resuspended in 50 μL sucrose solution. Cells were immediately used for electroporation. 2 μL of purified plasmid pJB3Tc20, pCMPG6231 or pCMPG6232 was added to the competent cells. These suspensions were transferred to 2 mm cuvettes (Eurogentec) and electroporated with a Biorad Gene Pulser, at 2.5 kV, 25 μF and 200 Ω. Subsequently, the electroporated cells were incubated for one hour at 37 °C prior to plating on LB supplemented with tetracycline.

### Phylogenetic analysis

Putative ColM-like proteins in *Burkholderia* genomes were identified using the National Center for Biotechnology (NCBI) non-redundant database, via Blast searches. Sequences of ColM catalytic domains in *E. coli, Pectobacterium* and *Pseudomonas* were used as search queries (Pfam PF14859). Similarly, *Burkholderia* genomes were searched for ColM-like immunity proteins (YebF-like proteins, Pfam PF13995). Multiple sequence alignments were generated with MUSCLE and phylogenetic analysis with PhyML, implemented in the Geneious v7.1.2. software. Amino-terminal signal sequences were predicted by PRED-TAT^42^ (http://www.compgen.org/tools/PRED-TAT). Transmembrane regions and topology were predicted by TMHMM (http://www.cbs.dtu.dk/services/TMHMM).

## Additional Information

**How to cite this article**: Ghequire, M. G. K. and De Mot, R. Distinct colicin M-like bacteriocin-immunity pairs in *Burkholderia. Sci. Rep.* **5**, 17368; doi: 10.1038/srep17368 (2015).

## Supplementary Material

Supplementary Information

## Figures and Tables

**Table 1:** Features of characterized and hypothetical BurM bacteriocins and cognate Bmi immunity proteins in *Burkholderia* species.

Strain	Origin	Accession number	BurM size (AA)	Signal peptide	BurM identity (%)	Bmi type	Bmi identity (%)	Accession number	Bmi size (AA)	*bmi* position
*B. ambifaria* MEX-5	Teosinte plant	EDT37562	379	Tat	─	A	─	EDT37561	110	U (−)
*B. glumae* PG1	Unknown	AJK48278	380	Tat	─	A	─	AJK48279	107	D (−)
*Burkholderia* sp. A1	Breeding environment of stag beetle	WP_051598391	373	Sec	─	A	─	NA	107	U (−)
*B. ambifaria* AMMD	Pea rhizosphere	ABI85884	372	Sec	─	B	─	ABI85883	124	D (+)
*B. cepacia* GG4 *B. pyrrocinia* CH-67 *Burkholderia* sp. A9	Ginger rhizosphere Forest soil Tropical soil	AFQ49511 NA KHK57657	373 374 373	/ Tat /	─ 81.3 99.2	B B B	─ 88.7 99.2	AFQ49512 WP_017333579 KHK57656	124 124 124	D (+) D (+) D (+)
*B. ubonensis* Bu	Soil	NA	379	Tat	─	B	─	WP_010092909	124	D (+)
*B. cepacia* ATCC 25416 *B. ambifaria* MC40-6 *B. contaminans* LMG 23361	Onion Cystic fibrosis patient Milk of sheep with mastitis	AIO23154 ACB62834 NA	376 377 377	Tat Tat Tat	─ 84.3 86.7	B B B	─ 77.3 75.8	AIO23727 ACB62833 KKL41098	132 131 127	D (+) D (+) D (+)
*B. oklahomensis* C6786 *B. oklahomensis* EO147	Infected human wound Human ocular ulcer	AJX34277 AIO69425	392 392	Tat Tat	─ 99.2	B B	─ 100	AJX33992 AIO69736	111 111	D (+) D (+)
*B. cepacia* DWS 37UF10B-2 *B. cepacia* DWS 16B-4 *B. cenocepacia* CEIB S5-1	Soil Unknown Agricultural soil	KGB93731 KGC02247 NA	359 359 359	Tat Tat Tat	─ 96.1 95.3	B B B	─ 84.3 82.5	NA NA NA	114 108 114	D (+) D (+) D (+)

Protein sizes in number of amino acids (AA) include Tat- or Sec-dependent secretory signal peptide sequences, if predicted. Highly similar BurM and Bmi subtypes are grouped and the pairwise AA sequence identity to a representative member of these groups (strains GG4, ATCC 25416, C6786, or DWS 37UF10B-2) is shown in percentage. The type of immunity protein (BmiA, BimB) is specified. The localization of the *bmi* genes is indicated by their position relative to the corresponding *burM* gene: downstream (D) or upstream (U); same strand (+) or opposite strand (−). NA, not annotated.

**Table 2:** Heterologous expression of *bmi* genes in burkhocin-susceptible strains.

Indicator strain	Immunity gene	BurM1 (MEX-5)	BurM2 (AMMD)
LMG 18830	Control	+	─
	*bmiA*_*MEX-5*_	─	─
	*bmiB*_*AMMD*_	+	─
LMG 18941	Control	+	T
	*bmiA*_*MEX-5*_	─	T
	*bmiB*_*AMMD*_	+	─
LMG 18943	Control	+	T
	*bmiA*_*MEX-5*_	─	T
	*bmiB*_*AMMD*_	+	─
LMG 24506	Control	+	─
	*bmiA*_*MEX-5*_	─	─
	*bmiB*_*AMMD*_	+	─

Selected Bcc strains were transformed with shuttle vector pJB3Tc20 (control, no insert), or with pJB3Tc20-derived plasmids equipped with different *bmi* genes, and tested for susceptibility to BurM1 and BurM2 via spot assay. +, clear halo; T, turbid halo: reduced cell density compared to cell lawn; −, no growth inhibition of the indicator strain.
