# Supplementary material for: Distinct colicin M-like bacteriocin-immunity pairs in Burkholderia
Source: Sci Rep. 2015 Nov 27;5:17368. doi: 10.1038/srep17368 (PMC4661593; doi:10.1038/srep17368)
Supplement: Supplementary Information [file srep17368-s1.pdf]

Distinct colicin M-like bacteriocin-immunity pairs in *Burkholderia*

Supplementary information

Maarten G. K. Ghequire*, René De Mot

Centre of Microbial and Plant Genetics, University of Leuven, 3001 Heverlee, Belgium

* Author of correspondence: Maarten G. K. Ghequire

e-mail: maarten.ghequire@biw.kuleuven.be
Tel: +32 16 376638
Fax: +32 16 321963

Fig. S1. Multiple sequence alignment of BurM proteins. Abbreviations for species names: Bamb, *Burkholderia ambifaria*; Bcen, *Burkholderia cenocepacia*; Bcep, *Burkholderia cepacia*; Bcon, *Burkholderia contaminans*; Bglu, *Burkholderia glumae*; Bokl, *Burkholderia oklahomensis*; Bpyr, *Burkholderia pyrrocinia*; Bubo, *Burkholderia ubonensis*; Burk, *Burkholderia* sp. Different shading indicates the degree of conservation. Predicted Sec signal sequences are boxed in red and Tat signal sequences in orange, the conserved motif at the amino-terminus in purple (see Sequence alignment and logo in Fig. 2), the ColM domain in yellow, conserved residues in the catalytic domain in green. The aspartate residue that was mutated in BurM2 is marked with an asterisk.

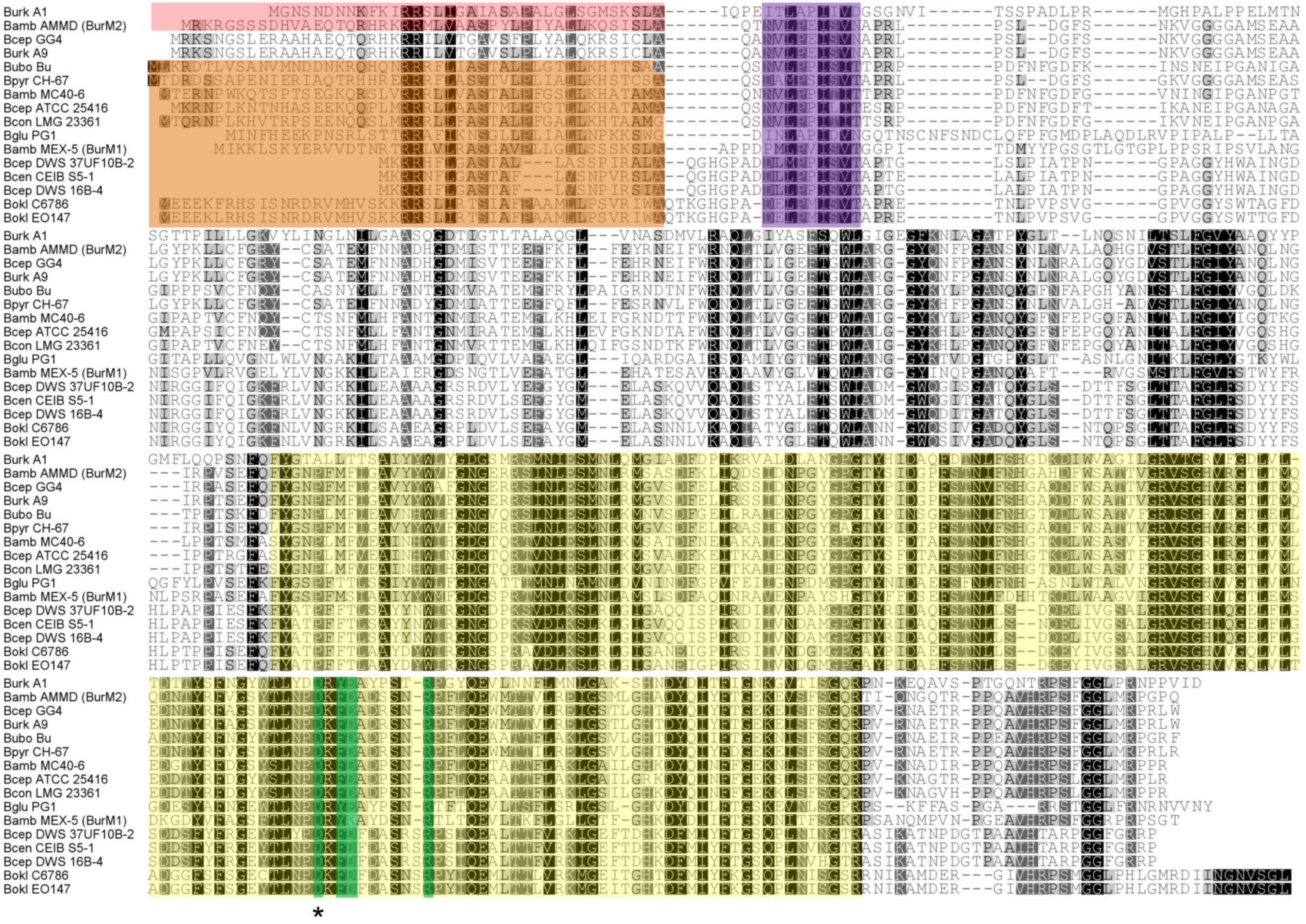
Burk A1
Bamb AMMD (BurM2)
Bcep GG4
Burk A9
Bubo Bu
Bpyr CH-67
Bamb MC40-6
Bcep ATCC 25416
Bcon LMG 23361
Bglu PG1
Bamb MEX-5 (BurM1)
Bcep DWS 37UF10B-2
Bcen CEIB S5-1
Bcep DWS 16B-4
Bokl C6786
Bokl EO147

Fig. S2. Purification of BurM proteins. SDS PAGE electrophoresis of purified BurM1 (lane 2), BurM2 (lane 3) and BurM2(D297A) (lane 4). Kaleidoscope ladder with size marks (kDa) is visible in line 1.

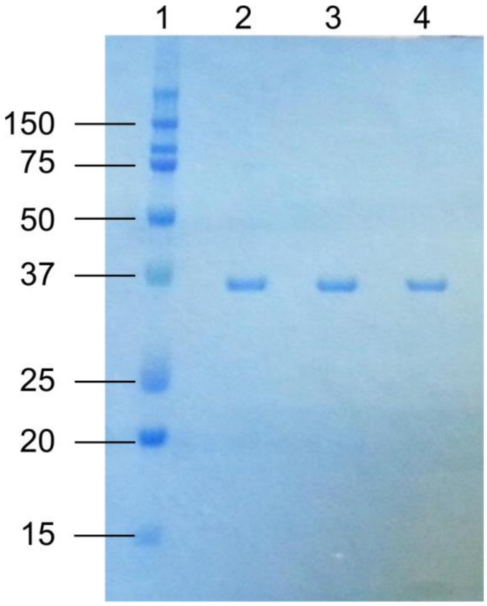

Fig. S3. Homology and phylogeny of BmiA proteins. Multiple sequence alignment (A) and unrooted maximum likelihood phylogenetic tree (B) of BmiA proteins. Encoded proteins without associated toxin (orphans) are marked with (o). Abbreviations for species names: Bpse, *Burkholderia pseudomallei*; Btha, *Burkholderia thailandensis*; other abbreviations as in Fig. S1. (A) Predicted transmembrane regions are boxed in blue and the conserved cysteine residue is colored green. Different grey shading indicates the degree of conservation. (B) Scale bar represents 0.3 substitutions per site. Bootstrap values (percentages of 1000 replicates) are shown at the branches.

A

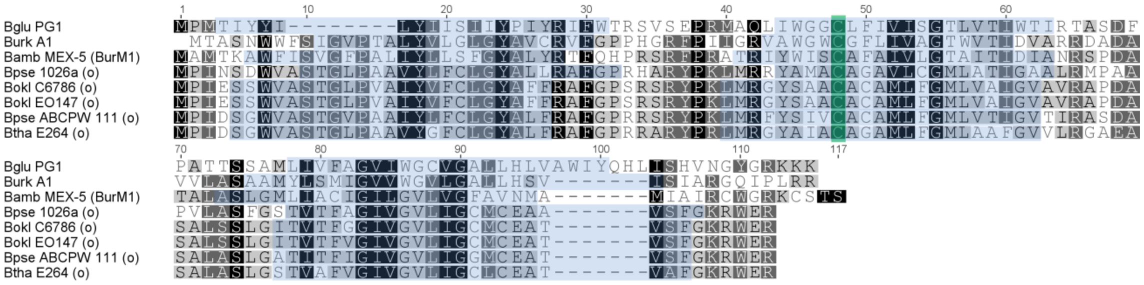

B

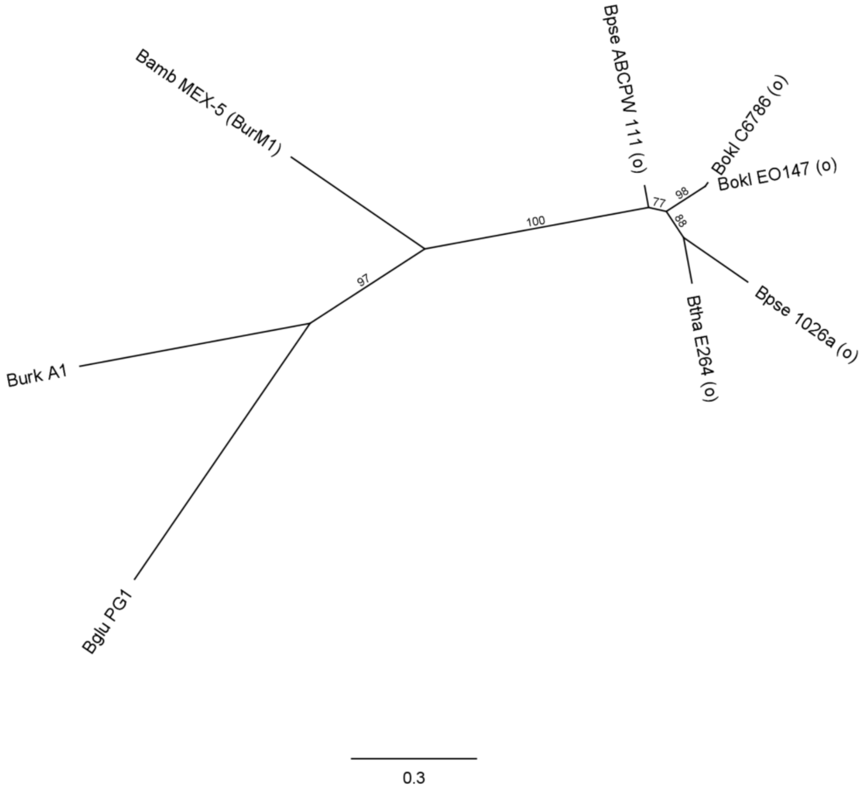

Fig. S4. Homology and phylogeny of BmiB proteins. Multiple sequence alignment (A) and unrooted maximum likelihood phylogenetic tree (B) of BmiB proteins. Encoded proteins without associated toxin (orphans) are marked with (o). Abbreviations for species names: Bmul, *Burkholderia multivorans*; other abbreviations as in Fig. S1. (A) Predicted transmembrane regions are boxed in blue, and predicted Sec-dependent signal peptide sequences in red. The conserved cysteine residues are highlighted in green boxes. Different shading indicates a different degree of conservation. (B) Scale bar represents 0.4 substitutions per site. Bootstrap values (percentages of 1000 replicates) are shown at the branches.

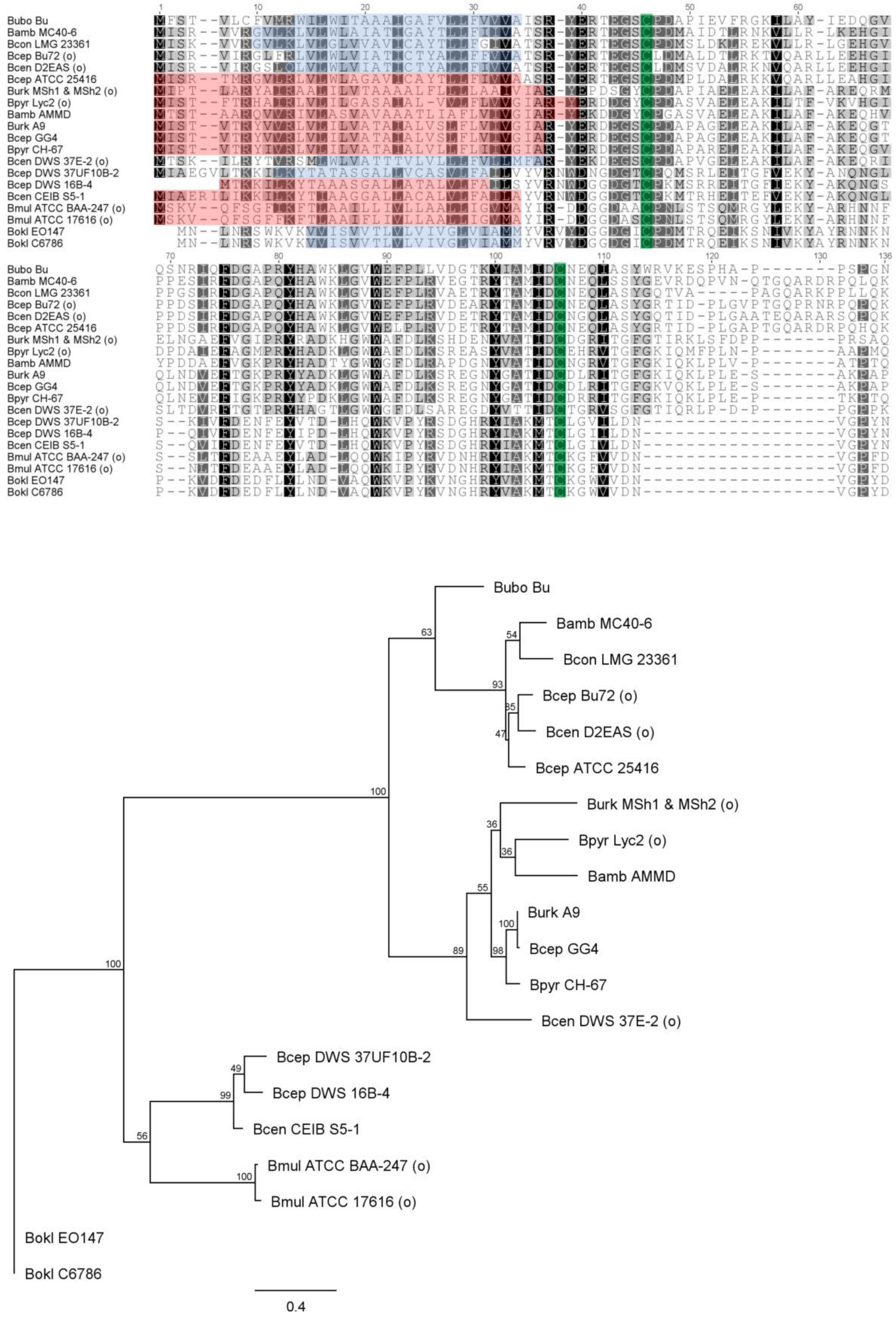

Table S1. Susceptibility patterns of *Burkholderia* strains to BurM1 and BurM2. +, clear halo; T, turbid halo: reduced cell density compared to cell lawn; -, no growth inhibition of the indicator strain; /, not informative.

Species	Strain number	BurM1	BurM2
B. ambifaria	LMG 17828	-	-
	LMG 17829	-	-
	LMG 19182 (or AMMD)	-	/
	LMG 19466	-	-
	LMG 19467	-	-
	LMG 26702 (or MEX-5)	/	-
B. anthina	LMG 20980	-	-
	LMG 20983	-	-
B. arboris	LMG 24066	-	-
B. cenocepacia	LMG 6986	-	-
	LMG 16656	-	-
	LMG 16659	+	-
	LMG 18826	-	-
	LMG 18827	-	-
	LMG 18828	-	-
	LMG 18829	+	+
	LMG 18830	+	-
	LMG 18863	-	-
	LMG 19230	-	-
	LMG 21461	+	-
	LMG 21462	+	-
	LMG 24506	-	-
B. cepacia	LMG 1222	-	-
	LMG 18821	-	-
B. contaminans	LMG 16227	+	-
B. diffusa	LMG 24065	-	-
	LMG 24266	-	-
B. dolosa	LMG 18941	+	T
	LMG 18943	+	T
B. lata	LMG 6992	-	-
B. latens	LMG 24064	-	-
B. metallica	LMG 24068	T	-
B. multivorans	LMG 13010	-	-
	LMG 18825	-	-
B. pyrrocinia	LMG 14191	T	-
	LMG 21824	-	-
B. seminalis	LMG 24067	+	-
	LMG 24272	-	-
B. stabilis	LMG 14086	-	-
	LMG 14294	-	-
B. ubonensis	LMG 20358	-	-
	LMG 24263	-	-
B. vietnamensis	LMG 10929	-	-
	LMG 18835	-	-

Table S2. List of primers and plasmids used in this study.

Primer/plasmid	Sequence[a]/charcteristics	Purpose of use/origin
PGPRB-8001	AAGTTGGGTAACGCCAGGGT	Sequencing of inserts in pUC18
PGPRB-8002	GCACCCCAGGCTTTACACTTT	Sequencing of inserts in pUC18
PGPRB-8461	GCTCACTCATTAGGCACCC	Sequencing of inserts in pJB3Tc20
PGPRB-10029	TGGCAGCAGCCAACTCAGCTT	Sequencing of inserts in pET28(a)
PGPRB-10030	TATAGGCGCCAGCAACCGCA	Sequencing of inserts in pET28(a)
PGPRB-10070	TGGCTA**CTCGAG**TGTCCCGCTAGGACGCGGAC	Cloning of *burM1* in pET28(a)
PGPRB-10072	TGGCTA**CCATGG**GACCTCCTGATCCTATGTTGCCCGT	Cloning of *burM1* in pET28(a)
PGPRB-10077	TGGCTA**CTCGAG**CTGGGGTCCGGGGCGCATAA	Cloning of *burM2* in pET28(a)
PGPRB-10080	TGGCTA**CCATGG**GAACCAATGTGTTGCCGGAAATTTC	Cloning of *burM2* in pET28(a)
PGPRB-10117	GAGATGTTCAATAACGCTGACCACGGCGACATGATCTC CACCACGGA	Removal of internal NcoI site in *burM2*
PGPRB-10118	TCCGTGGTGGAGATCATGTCGCCGTGGTCAGCGTTATTG AACATCTC	Removal of internal NcoI site in *burM2*
PGPRB-10141	TGGCTA**GCATGC**CCAGTAGACTGCGATCAGTGTC	Cloning of *bmiB* in pJB3Tc20
PGPRB-10142	TGGCTA**GGATCC**CCCTGTGAGCGGAGTCATT	Cloning of *bmiB* in pJB3Tc20
PGPRB-10167	TGGCTA**CTGCAG**TATTTGACAGCGAAGGAGATGCAAAA TGA	Cloning of *burM2* in pUC18
PGPRB-10168	TGGCTA**TCTAGA**ACAGGGCCAATGTGACACTGA	Cloning of *burM2* in pUC18
PGPRB-10179	TCCTACACGTTGAATCCAGCCAAATTCGACGCCGATAGC	Construction of a D297A mutant in *burM2*
PGPRB-10180	GCTATCGGCGTCGAATTTGGCTGGATTCAACGTGTAGGA	Construction of a D297A mutant in *burM2*
PGPRB-10214	TGGCTA**CTGCAG**ATGGATATTCGATCCCCAGGGGA	Cloning of *bmiA* in pJB3Tc20
PGPRB-10215	TGGCTA**TCTAGA**TTGGGTGGACGTCGTAGAGGAA	Cloning of *bmiA* in pJB3Tc20
pET28(a)	pBR322 origin, His-tag/thrombin/T7 tag ; Km^R	Novagen
pJB3Tc20	Broad-host-range cloning vector; Ap^R, Tc^R	[1]
pUC18	ColE1 replicon, *lacZ*, cloning vector; Ap^R	[2]
pCMPG6228	pET28(a) with 1013-bp PCR-amplified fragment containing BamMEX5DRAFT_6664 from *B. ambifaria* MEX-5 cloned in NcoI/XhoI	This study
pCMPG6229	pET28(a) with 983-bp PCR-amplified fragment containing BAMB_RS01670 from *B. ambifaria* AMMD cloned in NcoI/XhoI	This study
pCMPG6230	Mutant construct of pCMPG6229; D297A of the encoded BmiB	This study
pCMPG6231	pJB3Tc20 with 411-bp PCR-amplified fragment containing BamMEX5DRAFT_6663 from *B. ambifaria* MEX-5 cloned in PstI/XbaI	This study
pCMPG6232	pJB3Tc20 with 447-bp PCR-amplified fragment containing BAMB_RS01665 from *B. ambifaria* AMMD cloned in SphI/BamHI	This study

[a] Restriction sites incorporated in the primers are shown in bold: GGATCC, BamHI; CCATGG, NcoI; CTGCAG, PstI; GCATGC, SphI; TCTAGA, XbaI; CTCGAG, XhoI.

References

1 Blatny, J. M., Brautaset, T., Winther-Larsen, H. C., Haugan, K. & Valla, S. Construction and use of a versatile set of broad-host-range cloning and expression vectors based on the RK2 replicon. *Appl. Environ. Microbiol.* **63**, 370-379 (1997).

2 Yanisch-Perron, C., Vieira, J. & Messing, J. Improved M13 phage cloning vectors and host strains: nucleotide sequences of the M13mp18 and pUC19 vectors. *Gene* **33**, 103-119 (1985).
